# Muscle Wasting and Sarcopenia in Heart Failure—The Current State of Science

**DOI:** 10.3390/ijms21186549

**Published:** 2020-09-08

**Authors:** Alessia Lena, Markus S. Anker, Jochen Springer

**Affiliations:** 1 Division of Cardiology and Metabolism, Department of Cardiology, Charité-Campus Virchow Klinikum (CVK), 13353 Berlin, Germany; markus.anker@charite.de; 2 Department of Cardiology, Charité-Campus Benjamin Franklin (CBF), 12203 Berlin, Germany; 3 Berlin Institute of Health Center for Regenerative Therapies (BCRT), 10178 Berlin, Germany; jochen.springer@charite.de; 4 Deutsches Zentrum für Herz-Kreislauf-Forschung (DZHK) (German Centre for Cardiovascular Research), Partner Site, 10785 Berlin, Germany

**Keywords:** heart failure, sarcopenia, cardiac cachexia, treatment

## Abstract

Sarcopenia is primarily characterized by skeletal muscle disturbances such as loss of muscle mass, quality, strength, and physical performance. It is commonly seen in elderly patients with chronic diseases. The prevalence of sarcopenia in chronic heart failure (HF) patients amounts to up to 20% and may progress into cardiac cachexia. Muscle wasting is a strong predictor of frailty and reduced survival in HF patients. Despite many different techniques and clinical tests, there is still no broadly available gold standard for the diagnosis of sarcopenia. Resistance exercise and nutritional supplementation represent the currently most used strategies against wasting disorders. Ongoing research is investigating skeletal muscle mitochondrial dysfunction as a new possible target for pharmacological compounds. Novel agents such as synthetic ghrelin and selective androgen receptor modulators (SARMs) seem promising in counteracting muscle abnormalities but their effectiveness in HF patients has not been assessed yet. In the last decades, many advances have been accomplished but sarcopenia remains an underdiagnosed pathology and more efforts are needed to find an efficacious therapeutic plan. The purpose of this review is to illustrate the current knowledge in terms of pathogenesis, diagnosis, and treatment of sarcopenia in order to provide a better understanding of wasting disorders occurring in chronic heart failure.

## 1. Introduction

Sarcopenia is defined as a diminished muscle strength, rooted in a reduction of muscle quantity and quality, often associated with reduced physical performance, according to the new definition of the European Working Group on Sarcopenia in Older People [1]. Since muscle mass, strength, and function are strongly influenced by demographic and anthropometric features [2], worldwide uniformed threshold values have not been established yet. This limitation, in conjunction with other definitions adopted, leads inevitably to incongruities in the assessment of sarcopenia among different populations [3] (Table 1). Sarcopenia is commonly observed in older patients, with a prevalence between 10 and 40%, depending on the definition used and the age range used in the studies [4]. The percentage of muscle mass loss progressively increases over the years, starting from the 5th decade with 1%/year and reaching up to 50% by the 8th–9th decade of life [5]. Interestingly, a recent meta-analysis of 41 studies and 34,955 participants showed that the prevalence of sarcopenia in nursing home individuals in the included studies were much higher (51% (95% CI: 37–66%) in men and 31% (95% CI: 22–42%) in women) compared to community-dwelling individuals (11% (95% CI: 8–13%) in men and 9% (95% CI: 7–11%) in women), possibly due to lower activity levels in nursing homes [6].

Recent evidence suggests that a dysregulation of immunosenesence and low-grade progredient inflammatory response in elderly persons (inflammageing) [7,8] may be involved in the development of sarcopenia [9,10]. Diet and physical activity have been associated with inflammatory activation in age-related sarcopenia [11]. In addition, epigenetic mechanisms may be involved in age-related muscular changes [12]—a study comparing blood DNA methylation in sarcopenic and non-sarcopenic old women (>65 years) reported a lower methylation of differentially methylated cytosin–phosphate–guanine sites (dmCpGs) related to Kyoto Encyclopedia of Genes and Genomes (KEGG) signaling pathways associated with muscle function and energy metabolism in the sarcopenic group (*p* = 0.004), suggesting that these processes might be epigenetically altered in ageing sarcopenia. Hypermethylated promoter regions of genes associated with metabolism in the sarcopenic group also indicate a possible suppression of cellular energy regulation in these subjects. Muscle wasting represents a major risk factor for decreased muscular resistance [13] and loss of independency in daily life activities (19.6% vs. 13.8% of dependency, sarcopenia vs. non-sarcopenia, respectively, *p* < 0.001) [14]. In a recent meta-analysis using 33 studies with more than 45,000 individuals, it was shown that sarcopenia was significantly associated with bone fractures. Sarcopenic individuals had a significant higher risk of falls (cross-sectional studies: Odds Ratio (OR): 1.60, 95% CI: 1.37–1.86, *p* < 0.001, I2 = 34%; prospective studies: OR: 1.89, 95% CI: 1.33–2.68, *p* < 0.001, I2 = 37%) and fractures (cross-sectional studies: OR 1.84, 95% CI 1.30–2.62, *p* = 0.001, I2 = 91%; prospective studies: OR 1.71, 95% CI 1.44–2.03, *p* = 0.011, I2 = 0%) compared with non-sarcopenic individuals [15]. A study with 4452 disability-free adults aged ≥65 years investigating disability in sarcopenia (mean follow-up 30 months) found that compared to non-sarcopenia, individuals with sarcopenia or low serum albumin alone had an increased risk of disability (Hazard ratio (HR): 2.74, 95% CI: 1.58–4.77, and HR: 1.71, 95% CI: 1.26–2.33, respectively), which was further increased in the groups that had both sarcopenia and low serum albumin (HR: 3.73, 95% CI: 1.87–7.44) [16]. A prospective cohort of 534 individuals (73.5 ± 6.2 years, 60.5% female) [17] showed a higher mortality (16.2% vs. 4.6%, *p* < 0.001) of individuals diagnosed with sarcopenia than of those who were not diagnosed after 3 years, if no association between baseline sarcopenia and physical disabilities or institutionalizations was highlighted [18]. A small study comparing 30 sarcopenic vs. 30 control individuals (77 ± 6 years, and 58% females) showed that sarcopenia may be associated with reduced diaphragmatic muscle thickness and respiratory functions.

The correct assessment of sarcopenia still represents a challenge for clinicians. Whether dual-energy X-ray absorptiometry (DXA) scan should represent the current reference standard for the skeletal muscle measurement is still a matter of debate [19,20]. High costs and scarce availability of this technique have led to the search for alternatives. The recent development of the D3-diluition method [21,22] with high reproducibility and minimized invasiveness has accomplished promising results in the estimation of skeletal muscle mass but its adoption in the clinical setting as a routine method remains to be implemented. A robust panel of biomarkers to detect the first signs of muscular degradation has not been established yet. Another frequently seen co-morbidity in these patients is cachexia, which is also in itself often accompanied by reduced hand grip strength and/or low walking speed [23], as well as worse performance in the short physical performance battery test [24]. However, a lack of uniform reference values for sarcopenic patients in these tests strongly demand a standardization in the clinical assessment of sarcopenia [25]. Recently, in a study based on 469,830 UK Biobank participants, associations of sarcopenia with adverse outcomes (all-cause mortality, incidence and mortality from cardiovascular disease (CVD), respiratory disease, and chronic obstructive pulmonary disease (COPD)) were strongest when sarcopenia was defined as slow gait speed plus low muscle mass, followed by severe sarcopenia, strongly suggesting that this combination of physical capability markers should still be considered in the diagnosis of sarcopenia [26]. The Asian Working Group for Sarcopenia studied the prevalence of 2061 older community residents (>60 years of age) [27]. Comparing the AWGS2014 algorithm to the revised AWGS2019 algorithm [28] (slow gait speed cut-off at <1 m/s and prolonged five-time chair-stand time (≥12 s)), the authors identified 60 and 89 individuals with sarcopenia, respectively. Interestingly, the authors found a linear correlation between the severity of sarcopenia and carotid intima-media thickness (no sarcopenia: 0.94 ± 0.31, sarcopenia: 1.04 ± 0.41, and severe sarcopenia: 1.07 ± 0.55 mm, *p* = 0.003), which could be used as a new marker [29]. High levels of homocysteine (OR: 1.9, 95% CI: 1.0–3.6) and high sensitive C-reactive protein (hsCRP) (OR: 3.9, 95% CI: 2.2–6.9) were independently associated with sarcopenia in data of 1582 participants, with stronger correlations seen in women [30].

Sarcopenia can be a modifiable condition. A multimodal approach, based on physical activity [31,32] and dietary recommendation [33], seems currently to be the most effective strategy to counteract progressive age-dependent muscle impairments and improve quality of life as well as life expectancy. Recent evidence suggests that a protein intake above 1–1.5 g/kg/day may positively influence the anabolic–catabolic imbalance in subjects suffering from sarcopenia [34]. An association of dietary habits (7-day food record) in 254 men with a mean age of 71 at baseline with the prevalence of sarcopenia 16 years later was described [35]. A healthy dietary pattern based on the dietary guidelines defined by the WHO tended to protect against the development of sarcopenia over 16 years. In particular, the authors found indications that increased adherence to a Mediterranean dietary pattern might be advantageous. The authors of a recent review suggest that elderly individuals with sarcopenia should eat at least three servings of fish a week to reach the minimal daily intake of 4–4.59 g of omega 3, reaching the 50% of recommended daily allowance (RDA) in vitamin E and D. High biological value of proteins in 150 g of fish and its high available magnesium (20% of RDA in 150 g of fish) suggest fish as a “functional food” in sarcopenia [36]. It has been shown that the combination of malnutrition and sarcopenia showed a synergistically accumulated risk for death in a prospective analysis of 427 hospitalized old adults over 80 years [37]. A metabolic signature has been described in a cohort of 189 sarcopenic individuals in which levels of essential amino acids including lysine, methionine, phenylalanine, threonine, as well as branched-chain amino acids and choline were inversely correlated with sarcopenia. Furthermore, nicotine metabolites (cotinine and trans-3′-hydroxycotine) and vitamin B6 status were linked to one or more clinical and functional measures of sarcopenia [38].

Other studies are investigating the molecular mechanisms involved in mitochondrial function [39] that might be relevant for muscle homeostasis in older age and could represent a new target for pharmaceutical interventions. Recent findings in older mice attribute a certain importance to peroxisome proliferator-activated receptor gamma coactivator 1-alpha (PGC-1α), whose expression is enhanced by physical exercise, leading to increased oxidative phosphorylation (OXPHOS) protein levels in mitochondria beyond levels induced by exercise in wild type mice, while a muscle-specific PGC-1α knockout resulted in blunting the exercise-controlled increase in OXPHOS proteins [40]. A recent publication shows that humans with sarcopenia, independently of their ethnicity, reproducibly exhibit a prominent transcriptional signature of mitochondrial bioenergetic dysfunction as evidenced by low PGC-1α/ERRα signaling and downregulation of mitochondrial proteostasis genes. These changes result in fewer mitochondria, reduction of respiratory complex expression and activity, as well as low nicotinamide adenine dinucleotide (NAD+) levels due to its disturbed biosynthesis [41].

The protein kinase mechanistic target of rapamycin (mTOR) is also a crucial modulator for cell growth and its loss in skeletal muscles has been recently investigated in knockout mouse models, suggesting that mTOR activity is essential for the regulation of peroxisome proliferator-activated receptor (PPAR) and PPAR-gamma coactivator 1-alpha (PPAR/PGC-1α)-mediated OXPHOS capacity in vivo [42]. Furthermore, a mutant mTOR lacking the kinase activity induces robust suppression of postnatal muscle mammalian target of rapamycin complex 1 (mTORC1) signaling [42], demonstrating damaging effects of mTOR mutations in muscle metabolism. Surprisingly, mTORC1 is hyperactivated in sarcopenic muscle and a partial inhibition by novel compound (RAD001) resulted in an attenuation of sarcopenia shown by increased muscle mass and fiber type cross-sectional area, as well as downregulation of several genes associated with senescence. Hence, RAD001 may be considered a potential sarcopenia treatment [43].

Despite recent advances, the underlying mechanisms characterizing sarcopenia in ageing are still under investigation in preclinical as well as in clinical settings. Because of its negative impact on the quality of life it is necessary to increase the knowledge of this wasting process and to find preventive and therapeutic measures that may also be applied in patients with chronic diseases.

Therefore, the aim of this review is to increase clinical awareness of sarcopenia, with a particular focus on current pathogenetic knowledge and therapeutic possibilities that may counteract wasting disorders in chronic heart failure.

## 2. Sarcopenia in HF

Heart failure (HF) is a systemic disease afflicting up to 2% of the population worldwide [44,45]. Although it represents a major burden in terms of expenditure of socio-economic resources and costs [46], the successful accomplishments in diagnostics [47] and treatment [48,49,50] achieved in the last decades have led to an improvement in outcomes [51] and to an increased life expectancy [52]. Consequently, the number of older HF patients with increasing clinical complexity is progressively growing [53]. As a result, a multimodal approach is needed, combining many different medical disciplines to treat non-cardiac co-morbidities such as wasting disorders [54] and to lead to an improvement of different secondary outcomes as well [55,56]. Muscle wasting is one of the main causes for exercise intolerance and ventilatory inefficiency in HF patients [57]. It promotes the aggravation of other clinical conditions and causes a deterioration of quality of life [58]. It is associated with a longer hospital stay [59], more frequent re-hospitalizations [60], and worsened prognosis [61]. In the Studies Investigating Co-morbidities Aggravating Heart Failure (SICA-HF) [62], which enrolled 200 chronic HF patients, the prevalence of sarcopenia in HF patients with reduced ejection fraction (HFrEF) was nearly 20% higher than in healthy adults of the same age [62,63]. Similar results have been observed in HF patients with a preserved ejection fraction [64,65]. Therefore, sarcopenia and chronic HF seem to be intertwined, complicating the progression and outcome of each other [66]. Sarcopenia can even be found in obese HF patients (“sarcopenic obesity” [67]) with a prevalence between 1.3% and 17.5% [68]. Even though these patients show higher amounts of body fat, they have lower muscle mass [69]. Many different mechanisms can influence the muscle metabolism in HF patients such as hyper-activation of the sympathetic system, systemic inflammation, and an alteration of neuro-hormonal release [70]. Elevated oxidative processes, increased activity of the ubiquitin–proteasome system, higher apoptotic activity, and reduced release of the skeletal muscle growth factors contribute to a generalized catabolic shift in the muscular tissue homeostasis [71]. As a result of these alterations, a systemic enhanced protein degradation causes muscle wasting. It is primarily characterized by atrophy of the fast-twitching type II myofibers but also the slow-twitching type I myofibers, decreased muscular capillary density, and fat infiltration [72].

HF patients present with various hormonal disturbances [73]—impaired expression of insulin growth factor 1 (IGF-1) [74], vitamin D deficiency [75], reduced levels of testosterone [76], and reduced levels of growth hormone (GH) [77] have been reported. A cross-sectional study with 3276 elderly participants, with sarcopenia defined by the Asian Working Group on Sarcopenia diagnostic criteria, showed that the appendicular skeletal muscle mass was positively associated with gender and Body Mass Index (BMI), as well as with GH, testosterone, IGF-1, mechanical growth factor (MGF), urea nitrogen, creatinine, and Hb levels, but negatively associated with HDL-C (all *p* < 0.05). Using logistic multivariable regression analysis, the authors showed an independent association between IGF-1, MGF, BMI, and gender with appendicular skeletal muscle mass (all *p* < 0.05) [78]. Since the IGF-1/GH axis contributes to the preservation of skeletal muscle mass [72], its modulation by supplementation of these hormones has been hypothesized to treat sarcopenia in older adults [79], but there is still no robust evidence of beneficial effects [80].

Vitamin D deficiency is common in old age [81], and there is evidence that this condition enhances the risk of falls and declined physical performance [82]. Additionally, low levels of vitamin D have been associated with risk of HF in elderly individuals [83]. Its supplementation in adults aged 60 years and older has reported positive results, increasing muscle strength and performance [82]. However, its replacement in chronic HF patients has only demonstrated improvements in the inflammatory profile but not in the exercise capacity nor in outcomes [84,85].

Low endogenous testosterone may represent an independent risk factor for HF [86]. Experimental administration of testosterone as a possible strategy to counteract exercise intolerance and dyspnoea in chronic HF has been investigated, describing positive results regarding reduction of symptoms [87] and increase of exercise capacity [88] in HF patients. However, the safety of testosterone supplementation and its potential negative effects on the cardiovascular system [89] (i.e., ischemic stroke, acute coronary syndrome, myocardial ischemia, congestive heart failure, death from coronary disease) have to be further examined [90].

Even though many HF patients experience a reduced exercise tolerance, resistance training has been demonstrated to be a positive stimulus on muscle mass, muscle quality, and physical performance in patients with HF [91]. The combination with aerobic exercise seems to exert anti-atrophic [92] as well as anti-inflammatory effects [93]. In general, physical activity is beneficial to prevent wasting [94] and to improve quality of life and prognosis in these patients [95,96].

With regards to medical treatment of sarcopenia, supplementation of essential amino acids (8 g/day) have shown positive results regarding the physical performance, but did not increase absolute muscle mass in patients with stable chronic HF and severe loss of muscle mass [97]. Some standard HF medications have demonstrated potential benefits against muscle loss. Angiotensin II-converting enzyme inhibitors (ACE-Is), due to their anti-oxidative and anti-inflammatory effects, could have muscle protective effects [98]. In 1998, Vescovo et al. [99] reported in a small study in 16 HF patients that a 6-month treatment with enalapril (*n* = 8) or losartan (*n* = 8) improved exercise capacity. In 2003, a sub-analysis of the Studies of Left Ventricular Dysfunction (SOLVD) [100], including 1929 chronic HF patients, showed that patients taking enalapril had a 19% lower risk of developing cachexia. Whether ACE-Is are beneficial in healthy older people remains unclear—a sub-analysis of the Berlin Aging Study II (BASE-II) study including 838 community-dwelling, elderly people found similar muscle mass, strength, and function in the patients with vs. without ACE-I [101], whereas a double-blind randomized controlled trial in 130 participants ≥65 years with functional impairment showed better functional capacity after 20 weeks with ACE-I vs. placebo [102]. ACE-Is may also help in counteracting angiotensin II-dependent catabolic effects by modulating the GH/IGF-1 axis [103]. Blocking ACE and therefore the generation of Ang-II results in an upregulation of ACE2 expression and activity in skeletal muscle leading to increased levels of Ang1–7 and activation of its receptor (MasR), which contributes to an improved insulin sensitivity [104].

Beneficial effects of mineralocorticoid antagonists on skeletal muscle homeostasis have been postulated [105]. Despite some positive results on muscle quality in rats [106] and on exercise capacity in HF patients [107], Burton et al. [108] did not find an association between spironolactone and better physical function in a randomized placebo-controlled trial including 120 participants aged >64 years without HF.

Some studies suggest that beta-blockers may slow down wasting processes associated with increased sympathetic activation—in an analysis from the COPERNICUS trial [109], a double-blind, placebo-controlled randomized trial in 2289 patients with HF, carvedilol in comparison to placebo exhibited a 33% lower risk of weight loss >6% (95% CI: 14–48%, *p* = 0.002) during 24 months of follow-up. The retrospective analysis of the COPERNICUS data confirmed the results of the prospective phase II ACT-ONE trial [110], a randomized, double-blind, placebo-controlled phase II study, including 87 colorectal and non-small cell lung cancer patients with cachexia, showing that espindolol (also termed ACM-001) was associated with weight gain of 2.83 kg (95% CI: 1.00, 3.68) compared with a weight loss of 0.99 kg (95% CI: −3.97, 1.52) in the placebo group, increased lean body mass (1.76 kg (95% CI: 1.43, 3.18) compared with a gain of 0.57 kg (95% CI: −0.01, 1.71) in the placebo group (*p*  =  0.012), and improved hand grip strength (high dose −1.15 ± 0.7 kg, placebo −3.51 ± 0.8 kg change per 4 weeks; *p* = 0.0134). This effect was even more pronounced in a highly aggressive cancer cachexia rat model (Yoshida AH-130 hepatoma), in which treatment of espindolol at 3 mg/kg/day resulted in a prevention of the progressive loss of fat mass (−6 ± 2 g vs. −12 ± 1 g; *p* < 0.001); lean mass (+1 ± 10 g vs. −37 ± 2 g; *p* < 0.001) and body weight (+1 ± 13 g vs. −60 ± 2 g; *p* < 0.001) were stable. Most importantly, survival was significantly improved (HR: 0.29, 95% CI: 0.16–0.51, *p* < 0.001). Mechanistically, espindolol reduces catabolic signaling (reduced myostatin, ubiquitin proteasome system (UPS) activity, autophagy), while increasing anabolic signaling (Protein kinase B, Akt/mTOR) [111]. Previously, the effects of espindolol on muscle mass in 19-month-old rats have been investigated, where 3 mg/kg/day espindolol treatment over a period of 4 weeks increased body weight (+8.0 ± 6.1 g, *p* < 0.05), particularly lean mass (+43.4 ± 3.5 g, *p* < 0.001), and reduced fat mass (−38.6 ± 3.4 g, *p* < 0.001), while placebo rats progressively lost body weight (−15.5 ± 7.2 g), lean mass (−1.5 ± 4.2 g), and fat mass (−15.6 ± 2.7 g), thereby reversing the effects of sarcopenia [112].

Currently, some compounds for wasting disorders in chronic HF are being tested in preclinical and clinical settings [113]—acylated ghrelin has a potential anti-catabolic effect, as demonstrated by an experimental study conducted in a chronic HF rat model [114], possibly by regulation of the UPS rate-limiting E-3 ubiquitin ligases, muscle RING-finger protein-1 (MuRF-1) and Muscle Atrophy F-box (MAFbx)/atrogin-1 [115]. Moreover, its intravenous administration in a small cohort of HF patients underlined an amelioration of exercise capacity and muscle strength [116]. Anamorelin, a non-peptide ghrelin analogue, was recently tested in healthy young men [117], exhibiting gain in appetite, food intake, and weight. In non-small cell lung cancer patients [118], the same compound produced additional improvement in the lean body mass and in cachexia symptoms. Recently, a chronic HF mouse study showed diaphragm fiber atrophy, an 20% impaired contractile function, and reduced mitochondrial enzyme activities. Post left anterior descending artery-myocardial infarction (LAD-MI) treatment with the MuRF-1 inhibitor compound ID#704946 partially prevented the chronic HF effects on the diaphragm [119].

The negative regulator of muscle mass myostatin (also known as Growth/differentiation factor 8 (GDF-8)) binds primarily to the activin II B receptor (ActRIIB) and is upregulated under catabolic conditions such as sarcopenia and cachexia. The knockout of myostatine gene led to a significantly increased muscle mass in mice [120]. However, the relationship between muscle mass and strength in these mice was not linear. There are spontaneous, natural gene deletions in animals such as Belgian Blue cattle; whippets; and, in a rare case, humans [121]. Human myocardium expressed increased levels of myostatin in end-stage heart failure compared the control group. The related signaling pathways in the myocardium were seen to have a gender effect [122]. Myostatin expressed and secreted by the myocardium is thought to be causal for skeletal muscle wasting in a transaortic constriction chronic HF mouse model [123]. Binding of activin A to ActRIIB in skeletal muscle was shown to induce muscle atrophy that was dependent on a p38beta Mitogen-Activated Protein Kinase (MAPK)-activated signaling pathway and resulted in the upregulation of ubiquitin ligases MAFbx and UBR2 (E3alpha-II), as well as increases in LC3-II, a marker of autophagosome formation [124]. Plasma activin A levels have been reported to be an independent predictor of survival in cancer patients [125]. Interestingly, doxorubicin-induced cachexia was attenuated by ActRIIB ligand blocking. Pre-treatment with soluble ACVR2B-Fc had only a minor impact on the cardiac muscle while it showed strong effects in skeletal muscle at the transcriptome level [126]. These data should make myostatin blocking an interesting strategy to counteract muscle loss in various conditions and diseases, however, while neutralizing antibodies such as MYO-029, AMG 74, LY2495655, or soluble receptor decoys such as ACE-11 and ACE-031 have significant beneficial effects on muscle mass and strength, they also exhibit several side effects including urticaria, aseptic meningitis, diarrhea, confusion, fatigue, and unintentional muscle contractions [79].

Different selective androgen receptor modulators (SARMs) [127] are currently being explored due to their potential anabolic activity but without side effects of androgens. Enobosarm showed some promising results in a double-blind, placebo-controlled phase II trial, enrolling cancer patients with at least 2% weight loss in the 6 months before recruitment. A significant increase in total lean body mass over 4 months was observed in patients treated with 1 mg enobosarm once daily (median 1.5 kg (range 2.1–12.6 kg), *p* = 0.0012)) and 3 mg enobosarm (median 1.0 kg (range −4.8–11.5 kg), *p* = 0.046), while placebo resulted in no change (median 0.02 kg (range −5.8–6.7 kg), *p* = 0.88) [128]. Nonetheless, there was no improvement in muscle strength nor physical performance. GSK2881078 [129], another SARM compound, determined dose-dependent gain in lean mass in healthy subjects, but a major response was observed in postmenopausal women while MK-4541 [130], an androgen receptor agonist with 5α-reductase inhibitor function, exhibited anabolic effects and improvement of muscle function in castrated male mice. Despite the promising results, data from large-scale studies confirming the potential muscle-protective effects of these compounds in HF patients are not available yet.

Some of the mechanisms involved in muscular wasting such as mitochondrial dysfunction [131], overactivation of the ubiquitin–proteasome system [132], and abnormal cellular autophagy [66] are still under investigation and might be possible targets for future therapeutic options.

## 3. Sarcopenia in Cardiac Cachexia

A sarcopenic phenotype in patients may precede and present with cachexia in patients with advanced stages of HF [133], a condition associated with an extremely reduced survival [134]. Cachexia has been diagnosed in 19% of male patients with stable chronic HF, while 7% had both sarcopenia and cachexia [62]. Another study confirms that the prevalence of cachexia in chronic HF ranges from 10% [135] to 16% [136]. Cachexia seems to result in a progressive systemic tissue depletion, which involves the skeletal muscle and the fat tissue [137]. Clinically, it is defined by an unintentional weight loss of ≥5% in the last 12 months and three of the following five components: abnormalities in blood tests (increased inflammatory biomarkers, hemoglobin <12 g/dL, and serum albumin <3.2 g/dL), reduced muscular strength, anorexia, low fat-free mass index, and signs of fatigue [134]. It also occurs, under the common denominator of chronic inflammation [138,139], in other various chronic diseases, e.g., in chronic obstructive pulmonary disease (COPD) [140], chronic kidney disease (CKD) [141,142], and cancer [143].

Contributing elements to the deleterious changes in body composition in patients with cardiac cachexia are anorexia, malnutrition, intestinal congestion [144], and an inflammatory cytokine storm, which have also been described as common complications in severe HF [145,146]. High serum levels of adiponectin [147], a protein involved in the cellular energy control of several tissues, have been found in HF patients with cachexia, unrelated to their body mass index [148]. This alteration describes its potential role as a biomarker of body fat changes, tissue wasting [149], as well as a predictor of mortality [150,151] in these patients. Furthermore, an association between adiponectin resistance and peripheral muscle abnormalities was found in non-cachectic HF patients over 61 years [152].

Cardiac cachexia is also associated with myocardial atrophy in rodent models. One of the key regulators seems to be muscle-specific ring finger 1 (MuRF1) [153], an E3 ubiquitin ligase present in skeletal as well in cardiac muscle—experimental small molecule inhibition of apoptotic and ubiquitin–proteasome-dependent proteolysis showed promising results in reducing muscle atrophy and contractile dysfunction in rodents with cardiac cachexia [119].

The current literature does not provide evidence of available or experimental pharmacological agents able to prevent or delay the progression of cardiac cachexia. Some promising results on mitigating side effects of tumors on the heart and on the prognosis through HF medications derive from an experimental experience in rats with cancer cachexia [154].

In conclusion, more efforts are needed to establish a worldwide, standardized definition and assessment of ageing as well as disease-related sarcopenia. More attention has to be paid to the early recognition and staging of wasting processes in HF. Large-scale trials in HF patients are needed to establish the efficacy and safety profile of new agents.

## Figures and Tables

**Table 1:** Comparison table of major diagnostic criteria and most frequently adopted cut-off points for sarcopenia.

EWGSOP 2010		EWGSOP2 2018		AWGS 2019		SDOC 2018	
**Creuz-Jentoft et al. (2010) [155]**		**Creuz-Jentoft et al. (2019) [1]**		**Chen et al. (2020) [28]**		**Bhasin et al. (2020) [156]**	
“Progressive and generalized loss of skeletal muscle mass associated with low muscle strength or low physical performance.”		“Sarcopenia is identified by low muscle strength and confirmed by additional low muscle quantity. Low physical performance describes a severe status.”		“Age-related loss of muscle mass, associated with low muscle strength and/or low physical performance.”		“Low muscle strength defined by low grip strength and low physical performance defined by low usual gait speed should be included in the definition of sarcopenia.”	
**Low muscle mass**		**Low muscle strength**		**Loss of muscle mass**		**Low muscle strength**	
DXA	ASM/height^2^ < 7.26 kg/m^2^ in men ASM/height^2^ < 5.5 kg/m^2^ in women	Handgrip	<27 kg in men <16 kg in women	DXA	SM/height^2^ < 7.0 kg/m^2^ in men SM/height^2^ < 5.4 kg/m^2^ in women	Handgrip	<35.5 kg in men <20.0 kg in women
BIA	SM/height^2^ < 8.87 kg/m^2^ in men SM/height^2^ < 6.42 kg/m^2^ in women	Chair stand test	>15 s for 5 rises	BIA	SM//height^2^ < 7.0 kg/m^2^ in men SM/height^2^ < 5.4 kg/m^2^ in women		
**Low muscle strength**		**Low muscle quantity or quality**		**Low muscle strength**		**Low physical performance**	
Handgrip	<30 kg in men <20 kg in women	DXA, BIA	ASM < 20 kg in men ASM < 15 kg in women	Handgrip	<28 kg in men <18 kg in women	Gait speed	Cut points dependent on age, sex, race/ethnicity, and disease
			ASM/height^2^ < 7 kg/m^2^ in men ASM/height^2^ < 5.5 kg/m^2^ in women				
		MRI, CT	Fat infiltration in skeletal muscle				
**Low physical performance**		**Low physical performance**		**Low physical performance**			
SPPB	≤8 point score	Gait speed	≤0.8 m/s	6-m walk test	<1.0 m/s		
6-m gait speed	<1.0 m/s	TUG	≥20 s	SPPB	≤9 point score		
4-m gait speed	<0.8 m/s	SPPB	≤8 point score	Five-time chair stand test	≥12 s		
		400 m walk test	Non-completion or ≥6 min for completion				

ASM, appendicular skeletal muscle mass; SM; total skeletal muscle mass; AWGS, Asian Working Group for Sarcopenia; BIA, bioelectrical impedance analysis; CT, computed tomography; DXA, dual-energy X-ray absorptiometry; EWGSOP, European Working Group on Sarcopenia in Older People; MRI, magnetic resonance imaging; SDOC, Sarcopenia Definition and Outcomes Consortium; SM, total skeletal muscle mass; SPPB, short physical performance battery; TUG, timed-up-and-go-test.
